# Supplementary material for: Assessing Ethnic Inequalities in Diagnostic Interval of Common Cancers: A Population-Based UK Cohort Study
Source: Cancers (Basel). 2022 Jun 23;14(13):3085. doi: 10.3390/cancers14133085 (PMC9264889; doi:10.3390/cancers14133085)
Supplement: Supplementary file 1 [file cancers-14-03085-s001.zip › cancers-1742809-supplementary.pdf]

Supplementary File S1: Flowchart of exclusion process

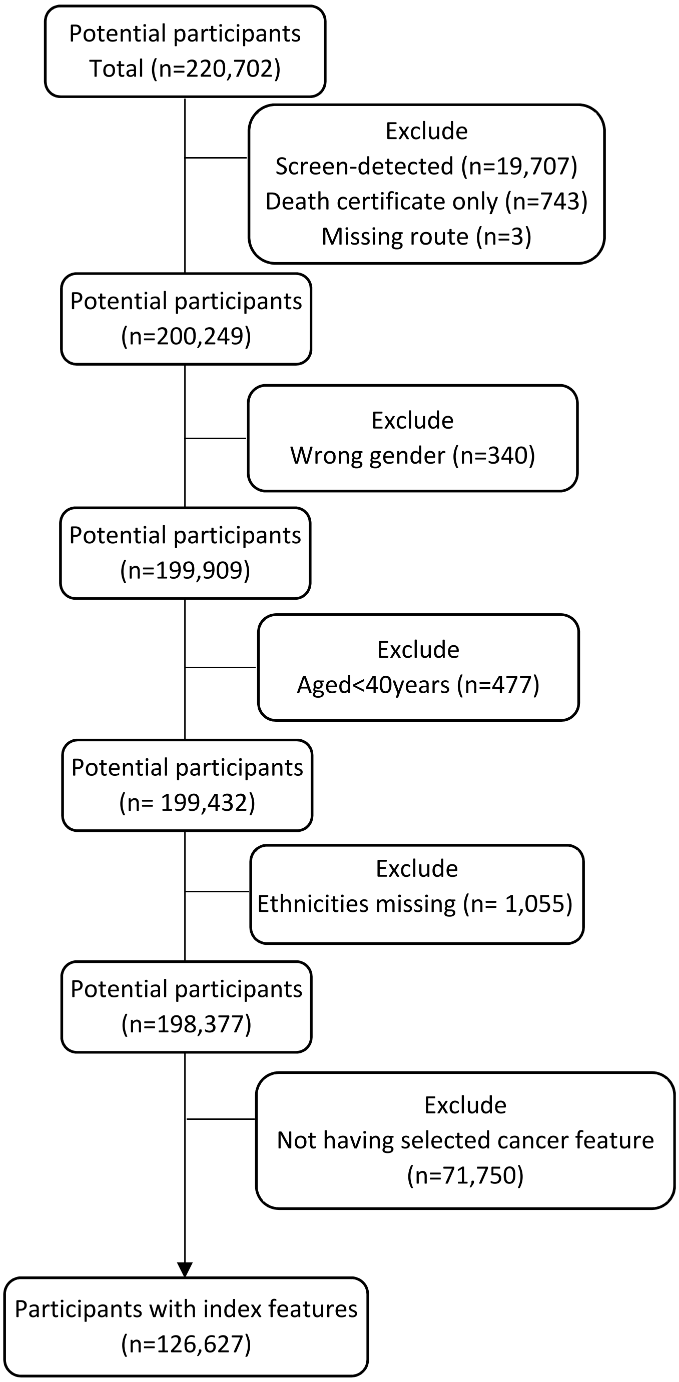

Supplementary File S2: Proportion of eligible participants with any of the selected cancer features (blue) or without the same (orange)

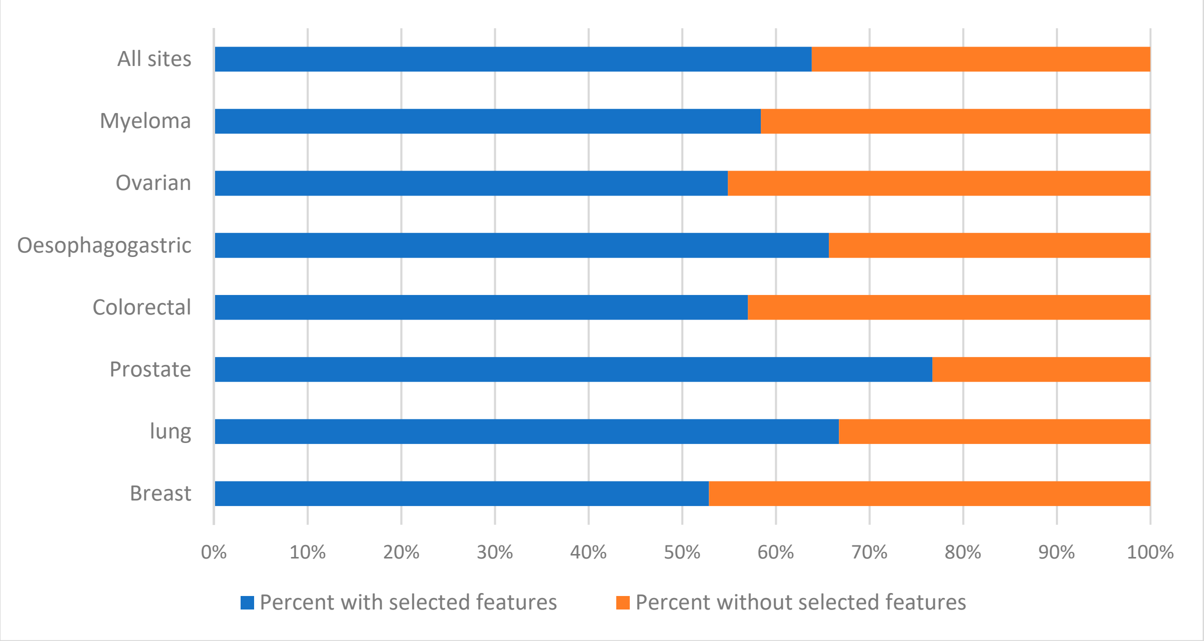

Supplementary File S3: recorded index feature of cancer by ethnicity

The Table below shows the number of patients with each cancer type (potential participants) and the number (and percentage) having relevant index feature based on the National Institute for Health and Care Excellence (NICE) guidance. Multiple index feature was common across all sites except in breast cancer, which was dominated by lump (18,517/20,002, 92.6%). For lung cancer, cough (24.4%), dyspnoea (22.2%), chest infection (15.1%), thrombocytosis (9.87%) and chest pain (7.37%) featured more frequently. Prostate cancer was characterised by raised PSA (70.4%), and lower urinary tract symptoms (20.5). Iron-deficiency anaemia (36.1%), abdominal pain (25.3%), rectal bleeding (22.3%) and change in bowel habit (11.0) were most frequent in colorectal cancer. For oesophagogastric cancer, dysphagia (23.4%), low haemoglobin/gastrointestinal bleeding (14.5%), dyspepsia (14.2%), upper abdominal pain (13.1%), acid reflux (9.25), and low back pain (7.77%) featured more frequently. Ovarian cancer was characterised by abdominal pain (35.9%), abdominal distention (9.84%), low back pain (9.68%), abdominal mass (8.06) and constipation (7.88%), while myeloma featured low back pain (34.8%), abnormal white blood cell count (21.1%) and abnormal erythrocyte sedimentation rate (20.6%). The pattern of recorded index feature by site (i.e., the most common feature(s)) was similar across ethnic groups, though the proportion vary slightly for myeloma and oesophagogastric cancer.

Recorded index feature of cancer by ethnicity

Sites	Feature	Participants with index feature					
		**All, n (%)**	**White, n (%)**	**Black, n (%)**	**Asian, n (%)**	**Mixed, n (%)**	**Other, n (%)**
**Breast (N=37,837)**	Breast pain	1,022 (5.11)	906 (4.96)	31 (8.20)	35 (6.51)	31 (7.03)	19 (5.21)
	Breast lump	18,517 (92.6)	16,945 (92.7)	340 (89.9)	495 (92.0)	399 (90.5)	338 (92.6)
	Breast skin changes (peau d'orange)	97 (0.48)	90 (0.49)	1 (0.26)	1 (0.19)	3 (0.68)	2 (0.55)
	Nipple discharge	269 (1.34)	245 (1.34)	6 (1.59)	6 (1.12)	7 (1.59)	5 (1.37)
	Nipple Retraction	70 (0.36)	74 (0.35)	0 (0)	0 (0)	1 (0.23)	1 (0.27)
	Lymphadenopathy (axilla)	27 (0.13)	26 (0.14)	0 (0)	1 (0.19)	0 (0)	0 (0)
	Total	**20,002 (52.9)**	**18,280 (91.4)**	**378 (1.89)**	**538 (2.69)**	**441 (2.20)**	**365 (1.82)**
**Lung (N=44,915)**	Appetite loss	197 (0.66)	175 (0.63)	7 (2.48)	4 (1.08)	2 (0.43)	9 (0.96)
	Chest infection	4,540 (15.1)	4,252 (15.2)	32 (11.4)	49 (13.2)	58 (12.6)	149 (15.9)
	Chest pain	2,210 (7.37)	2,056 (7.36)	18 (6.38)	44 (11.9)	34 (7.39)	58 (6.18)
	Chest signs consistent with lung cancer	290 (0.97)	269 (0.96)	4 (1.42)	3 (0.81)	5 (1.09)	9 (0.96)
	Cough	7,321 (24.4)	6,819 (24.4)	85 (30.1)	118 (32.8)	86 (18.7)	213 (22.7)
	Dyspnoea	6,649 (22.2)	6,243 (22.4)	51 (18.1)	50 (13.5)	132 (28.7)	173 (18.4)
	Fatigue	963 (3.21)	911 (3.26)	12 (4.26)	10 (2.70)	13 (2.83)	17 (1.81)
	Features suggestive of lung metastases	634 (2.11)	583 (2.09)	7 (2.48)	9 (2.43)	11 (2.39)	24 (2.56)
	Finger clubbing	56 (0.19)	47 (0.17)	0 (0)	3 (0.81)	0 (0)	6 (0.64)
	Haemoptysis	907 (3.03)	836 (2.99)	15 (5.32)	14 (3.77)	17 (3.70)	25 (2.66)
	Hoarseness	254 (0.85)	237 (0.85)	4 (1.42)	4 (1.08)	1 (0.22)	8 (0.85)
	Lymphadenopathy (supraclavicular, cervical)	101 (0.34)	93 (0.33)	1 (0.35)	0 (0)	0 (0)	7 (0.75)
	Shoulder pain	986 (3.29)	896 (3.21)	16 (5.67)	17 (4.58)	22 (4.78)	35 (3.73)
	Signs of superior vena cava obstruction	39 (0.13)	35 (0.13)	0 (0)	0 (0)	1 (0.22)	3 (0.32)
	Stridor	4 (0.01)	4 (0.01)	0 (0)	0 (0)	0 (0)	0 (0)
	Thrombocytosis	2,958 (9.87)	2,744 (9.83)	13 (4.61)	27 (7.28)	44 (9.57)	130 (13.8)
	Weight loss	878 (2.93)	805 (2.88)	10 (3.55)	9 (2.43)	16 (3.48)	38 (4.05)
	X-ray findings suggestive of lung cancer	991 (3.31)	921 (3.30)	7 (2.48)	10 (2.70)	18 (3.91)	35 (3.73)
	Total	**29,978 (66.7)**	**27,926 (93.2)**	**282 (0.94)**	**371 (1.24)**	**460 (1.53)**	**939 (3.13)**
**Prostate (N=47,508)**	Abnormal digital rectal examination	141 (0.39)	131 (0.39)	3 (0.25)	3 (0.52)	1 (0.13)	3 (0.47)
	Erectile dysfunction	1,492 (4.09)	1,305 (3.92)	98 (8.13)	36 (6.29)	37 (4.76)	16 (2.49)
	Haematuria, visible	1,694 (4.65)	1,559 (4.69)	45 (3.73)	29 (5.07)	31 (3.99)	30 (4.67)
	Lower urinary tract symptoms	7,475 (20.5)	6,880 (20.7)	212 (17.6)	115 (20.1)	152 (19.6)	116 (18.1)
	Raised PSA above age-specific value	25,650 (70.4)	23,381 (70.3)	847 (70.3)	389 (68.0)	556 (71.6)	477 (74.3)
	Total	**36,452 (76.7)**	**33,256 (91.2)**	**1,205 (3.31)**	**572 (1.57)**	**777 (2.13)**	**642 (1.72)**
**Colorectal (N=38,857)**	Abdominal mass	187 (0.84)	173 (0.84)	3 (0.96)	1 (0.29)	3 (0.77)	7 (1.34)
	Abdominal pain	5,612 (25.3)	5,186 (25.2)	91 (29.3)	93 (27.2)	95 (24.5)	147 (28.1)
	Change in bowel habit	2,442 (11.0)	2,307 (11.2)	18 (5.79)	19 (5.56)	44 (11.3)	54 (10.3)
	Faecal occult blood	97 (0.44)	88 (0.43)	2 (0.64)	2 (0.58)	3 (0.77)	2 (0.38)
	Iron-deficiency anaemia	7,995 (36.1)	7,433 (36.1)	112 (36.0)	135 (39.5)	132 (34.0)	183 (34.9)
	Rectal bleeding	4,929 (22.3)	4,584 (22.3)	72 (23.2)	81 (23.7)	96 (24.7)	96 (18.3)
	Rectal mass	81 (0.37)	77 (0.37)	2 (0.64)	0 (0)	0 (0)	2 (0.38)
	Weight loss	808 (3.65)	738 (3.58)	11 (3.54)	11 (3.22)	15 (3.87)	33 (6.30)
	**Total**	**22,151 (57.0)**	**20,586 (93.1)**	**311 (1.40)**	**342 (1.54)**	**388 (1.75)**	**524 (2.37)**

**Oesophago-gastric (N=16,640)**	Back pain	849 (7.77)	784 (7.76)	15 (8.62)	16 (9.94)	11 (8.15)	23 (6.48)
	Dyspepsia	1,549 (14.2)	1,434 (14.2)	23 (13.2)	17 (10.6)	17 (12.6)	58 (16.3)
	Dysphagia	2,551 (23.4)	2,380 (23.6)	23 (13.2)	38 (23.6)	29 (21.5)	81 (22.8)
	Haematemesis	152 (1.39)	140 (1.39)	7 (4.02)	1 (0.62)	2 (1.48)	2 (0.56)
	Low haemoglobin/gastrointestinal bleeding	1,588 (14.5)	1,445 (14.3)	37 (21.2)	30 (18.6)	26 (19.3)	50 (14.1)
	Nausea	208 (1.90)	201 (1.99)	2 (1.15)	1 (0.62)	2 (1.48)	2 (0.56)
	Reflux	1,011 (9.25)	949 (9.39)	7 (4.02)	12 (7.45)	13 (9.63)	30 (8.45)
	Suspicious barium meal results	6 (0.05)	5 (0.05)	1 (0.57)	0 (0)	0 (0)	0 (0)
	Thrombocytosis	635 (5.81)	592 (5.86)	6 (3.45)	8 (4.97)	8 (5.93)	21 (5.92)
	Upper abdominal mass	6 (0.05)	3 (0.03)	1 (0.57)	0 (0)	0 (0)	2 (0.56)
	Upper abdominal pain	1,436 (13.1)	1,306 (12.9)	31 (17.8)	35 (21.7)	18 (13.3)	46 (12.9)
	vomiting	399 (3.65)	366 (3.62)	8 (4.60)	2 (1.24)	3 (2.22)	20 (5.63)
	Weight loss	537 (4.91)	497 (4.92)	13 (7.47)	1 (0.62)	6 (4.44)	20 (5.63)
	Total	**10,927 (65.7)**	**10,102 (92.5)**	**174 (1.59)**	**161 (1.47)**	**135 (1.24)**	**355 (3.25)**
**Ovary (N=7,169)**	Abdominal distension/bloating	387 (9.84)	357 (9.80)	4 (12.5)	10 (10.6)	10 (10.8)	9 (9.09)
	Abdominal mass	317 (8.06)	298 (8.18)	1 (3.13)	8 (8.51)	4 (6.15)	6 (6.06)
	Abdominal pain	1,415 (35.9)	1,307 (35.9)	15 (46.9)	37 (39.4)	26 (40.0)	30 (30.3)
	Appetite loss	31 (0.79)	28 (0.77)	1 (3.13)	0 (0)	1 (1.54)	1 (1.01)
	Ascites	142 (3.61)	131 (3.59)	0 (0)	1 (1.06)	2 (3.08)	8 (8.08)
	Back pain	381 (9.68)	359 (9.85)	0 (0)	11 (11.7)	3 (4.62)	8 (8.08)
	Change in bowel habit	119 (3.02)	111 (3.05)	0 (0)	1 (1.06)	1 (1.54)	6 (6.06)
	Constipation	310 (7.88)	286 (7.85)	2 (6.25)	7 (7.45)	6 (9.23)	9 (9.09)
	Fatigue	180 (4.58)	162 (4.45)	7 (21.9)	6 (6.38)	3 (4.62)	2 (2.02)
	Pelvic mass	240 (6.10)	217 (5.95)	0 (0)	6 (6.38)	6 (9.23)	11 (11.1)
	Pelvic pain	73 (1.86)	70 (1.92)	1 (3.13)	1 (1.06)	1 (1.54)	0 (0)
	Raised Ca125	161 (4.09)	149 (4.09)	0 (0)	4 (4.26)	4 (6.15)	4 (4.04)
	Urinary urgency	12 (0.31)	12 (0.33)	0 (0)	0 (0)	0 (0)	0 (0)
	Urinary frequency	84 (2.14)	81 (2.22)	1 (3.13)	0 (0)	1 (1.54)	1 (1.01)
	Weight loss	82 (2.08)	76 (2.09)	0 (0)	2 (2.13)	0 (0)	4 (4.04)
	Total	**3,934 (54.9)**	**3,644 (92.6)**	**32 (0.81)**	**94 (2.39)**	**65 (1.65)**	**99 (2.52)**
**Myeloma (N=5,451)**	Bone pain	79 (2.48)	75 (2.64)	1 (0.71)	1 (1.23)	1 (1.82)	1 (1.64)
	Back pain	1,108 (34.8)	990 (34.8)	38 (27.1)	29 (35.8)	25 (45.5)	26 (42.6)
	Bence-Jones protein	176 (5.53)	156 (5.48)	10 (7.14)	3 (3.70)	2 (3.64)	5 (8.20)
	Abnormal erythrocyte sedimentation rate	657 (20.6)	576 (20.2)	34 (24.3)	24 (29.6)	12 (21.8)	11 (18.0)
	Hypercalcaemia	194 (6.09)	174 (6.11)	10 (7.14)	4 (4.94)	4 (7.27)	2 (3.28)
	Abnormal white cell count	673 (21.1)	600 (21.1)	40 (28.6)	15 (18.5)	6 (10.9)	12 (19.7)
	Pathological fracture	18 (0.57)	17 (0.60)	0 (0)	1 (1.23)	0 (0)	0 (0)
	Paraprotein	89 (2.80)	76 (2.67)	5 (3.57)	3 (3.70)	3 (5.45)	2 (3.28)
	Plasma viscosity consistent with myeloma	182 (5.72)	176 (6.18)	2 (1.43)	1 (1.23)	2 (3.64)	1 (1.64)
	Spinal cord compression due to myeloma	7 (0.22)	6 (0.21)	0 (0)	0 (0)	0 (0)	1 (1.64)
	Total	**3,183 (58.4)**	**2,846 (89.4)**	**140 (4.40)**	**81 (2.54)**	**55 (1.73)**	**61 (1.92)**
**All (N=198,377)**	-	**126,627 (63.5)**	**116,640 (92.1)**	**2,522 (1.99)**	**2,159 (1.71)**	**2,321 (1.83)**	**2,985 (2.36)**

Total, n (%) - the number and percentage of potential participants with index features
